# Supplementary material for: The causal role of circulating inflammatory markers in osteoporosis: a bidirectional Mendelian randomized study
Source: Front Immunol. 2024 Jul 18;15:1412298. doi: 10.3389/fimmu.2024.1412298 (PMC11291241; doi:10.3389/fimmu.2024.1412298)
Supplement: Supplementary file 1 [file DataSheet_1.zip › supplementary material/OSTEOPOROSIS-CIM/GCST90274833.tsv.gz_funnel.pdf]

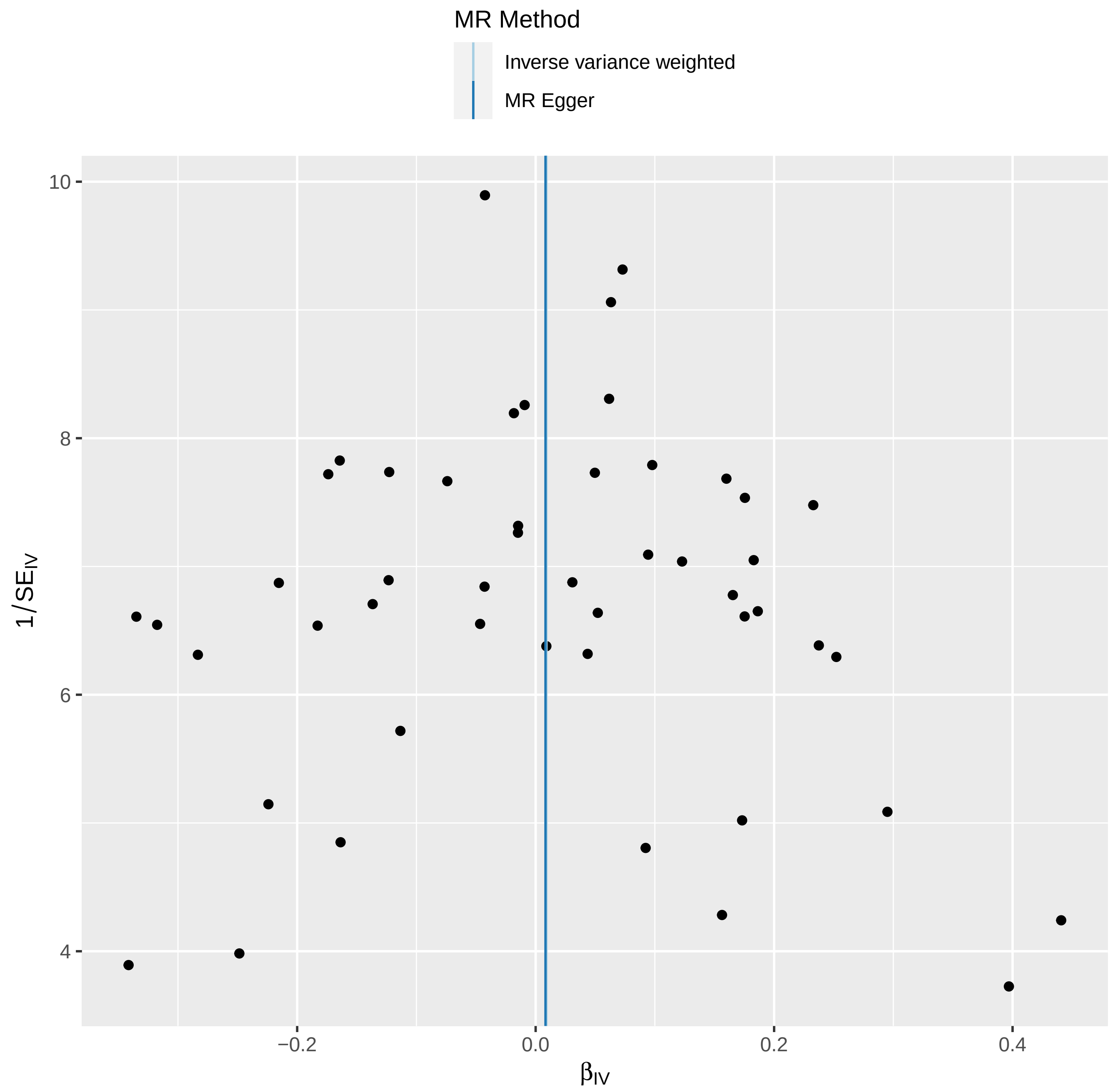
MR Method
Inverse variance weighted
MR Egger
10
8
6
4
1/SE_IV
−0.2
0.0
0.2
0.4
β_IV
